# A novel regulatory event-based gene set analysis method for exploring global functional changes in heterogeneous genomic data sets

**DOI:** 10.1186/1471-2164-10-26

**Published:** 2009-01-16

**Authors:** Chien-Yi Tung, Chih-Hung Jen, Ming-Ta Hsu, Hsei-Wei Wang, Chi-Hung Lin

**Affiliations:** 1 Institute of Microbiology and Immunology, National Yang-Ming University, Taipei, Taiwan; 2 VGH Yang-Ming Genome Research Center, Taipei, Taiwan; 3 Institute of Biochemistry and Molecular Biology, National Yang-Ming University, Taipei, Taiwan; 4 Medical Research & Education Division, Taipei City Hospital, Taipei, Taiwan

## Abstract

**Background:**

Analyzing gene expression data by assessing the significance of pre-defined gene sets, rather than individual genes, has become a main approach in microarray data analysis and this has promisingly derive new biological interpretations of microarray data. However, the detection power of conventional gene list or gene set-based approaches is limited on highly heterogeneous samples, such as tumors.

**Results:**

We developed a novel method, the regulatory **e**vent-based **G**ene **S**et **A**nalysis (eGSA), which considers not only the consistently changed genes but also every gene regulation (event) of each sample to overcome the detection limit. In comparison with conventional methods, eGSA can detect functional changes in heterogeneous samples more precisely and robustly. Furthermore, by utilizing eGSA, we successfully revealed novel functional characteristics and potential mechanisms of very early hepatocellular carcinoma (HCC).

**Conclusion:**

Our study creates a novel scheme to directly target the major cellular functional changes in heterogeneous samples. All potential regulatory routines of a functional change can be further analyzed by the regulatory event frequency. We also provide a case study on early HCCs and reveal a novel insight at the initial stage of hepatocarcinogenesis. eGSA therefore accelerates and refines the interpretation of heterogeneous genomic data sets in the absence of gene-phenotype correlations.

## Background

In the past decade microarray technology has become a popular tool for identifying differentially expressed genes (DEGs) associated with a given phenotype or sample classification. The biological interpretation of transcriptomic changes is commonly revealed by gene function annotation enrichment analysis based on a list of statistically selected DEGs. It is also called as Individual Gene Analysis (IGA) by Nam [1]. However, since only the most significant portion of DEGs was taken into account, the small set of genes might not perfectly represent the whole transcriptomic changes [1-5]. Indeed, Pan *et al*. showed that the selections of DEGs significantly affected the IGA results [6]. In addition, IGA usually tests functional changes by annotation enrichment methods such as hypergeometric test. These algorithms equally count each DEG so that the significance levels of gene-phenotype correlation are flattened [2].

Recently, gene set-based approaches have been proposed to overcome the drawbacks of IGA [1,3,7]. In principle, these approaches measure the gene-phenotype correlations (e.g. *t*-statistic) of every gene in a predefined gene set (GS), usually according to the Gene Ontology (GO) categories or the sets of genes representing biological pathways in the cell, and then give a GS score to represent its changes associated with the phenotype [1]. The statistical significance of the GS score can be determined by two different null hypotheses: competitive (*Q*_1_) and self-contained (*Q*_2_). In *Q*_1 _test, it assumes the genes in a gene set have the same level of association with the phenotype compared with the rest of the genes; in *Q*_2 _test, it assumes no gene in the gene set is associated with the phenotype. Although the gene set-based approaches are promising in deriving new information, their limitations and the underlying hypotheses have been discussed intensively. For example, *Q*_1 _approaches are very sensitive to correlation structure in gene sets that tend to give false positives [7]. *Q*_2 _approaches have low power of detection in highly varying samples such as clinical data sets [3]. Moreover, in *Q*_2 _test, it can be detected as significant by chance when the size of the gene set is large. Another approach, called gene set enrichment analysis (GSEA), which is a combination of two hypotheses (mixed model, *Q*_3_) has become one of the most mentioned and used gene set-based approaches [5,8]. However, a series of reports have also shown that GSEA inherited the limitations from both null hypotheses [3,8,9].

**Figure 1:**
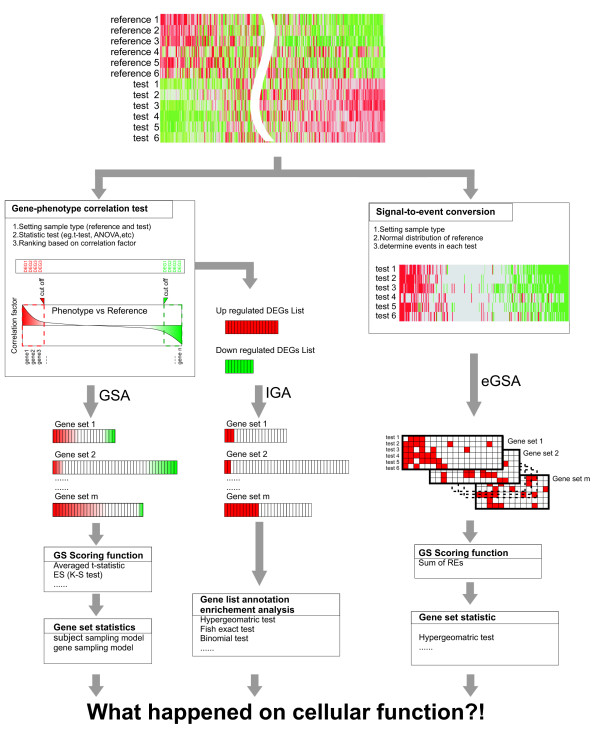
**Schematic representation of the analysis principles and differences between eGSA and other array analysis approaches**. Both individual gene analysis (IGA) and Gene Set Analysis (GSA) approaches are based on the gene-phenotype correlation. In GSA, the correlation value is mapped to GSs, and then summarized by the scoring functions such as averaged t-statistic or K-S test [5]. The significance of each score is then estimated by statistic tests [1]. In IGA, differentially expressed genes (DEGs) are selected based on the correlation threshold, and GS significance is estimated by enrichment statistics. In eGSA, the gene expression levels of test samples (test 1–6) are converted to expression regulatory events (REs) by comparing with the reference sample pool (reference 1–6). The sum of RE frequency is then used for GS scoring and estimation of the significant levels.

Although many gene set-based approaches and gene-based approaches have been developed, all of them are based on the same rationale, i.e., the detection of gene-phenotype correlation (Figure 1). This rationale is based on the assumption that samples with the same phenotypic change are homogenous and their gene expressions are correlated with their phenotypes. Such assumption is quite applicable for many cases, such as treatment versus control cell culture studies, but perhaps not always suitable for heterogeneous clinical samples, such as tumors versus normal tissues. Tumors are usually defined according to their common morphological and functional characteristics, such as abnormal growth, immortality, invasion etc. However, it is known that tumors have heterogeneous gene expression level even though they originated from the same tissue type and stage. This heterogeneity implies that those common characteristics could be regulated by various mechanisms. Thus, the functional changes might not be revealed by gene-phenotype correlation tests, and most gene-/gene set-based approaches based on this rationale cannot be applied on such data sets.

Here, we propose a novel method, called regulatory **e**vent-based **G**ene **S**et **A**nalysis (eGSA), to evaluate the significantly changed functions or pathways from diverse genomic data sets. eGSA is not based on the gene-phenotype correlations, but rather on gene expression regulatory events which are determined by comparing each gene expression level with the corresponding reference data set (Figure 1). Total regulatory events in a given gene set (GS) are counted as a GS score and then the significance of GS is tested by *Q*_1 _null hypothesis using hypergeometric test. Comparing with the approaches based on gene-phenotype correlation, we show in this study that eGSA can successfully derive new functional information from gene expression profiles that are very heterogeneous in nature.

## Results and discussion

### Heterogeneity of tumor transcriptome

**Figure 2:**
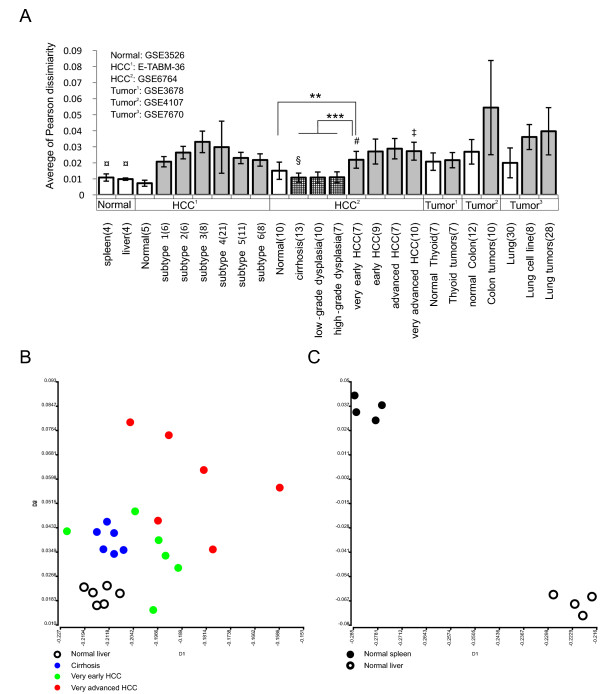
**Transcriptome heterogeneity of tumors**. (**A**) Six independent microarray experiments, including one normal tissue (Normal) and five tumor collections (HCC^1^, HCC^2^, tumor^1^, tumor^2^, and tumor^3^), were compared. The transcriptomic heterogeneity of each sample type was measured by the average of Pearson dissimilarity between paired samples. The heterogeneity of HCCs, even at the most initial stage, appeared to be significantly higher than those of normal livers (*p *= 1.73E-5, **) and pre-cancers (*p *= 3.38E-9, ***). Four data sets used for later analyses are marked (¤: ***Sts***, §***Sci***, #: ***Sve ***and ‡: ***Sva***), respectively. (**B**) The MDS plot of the normal liver and three different liver pathological sample sets. Both very early HCC (green circle) and very advanced HCC samples (red circle) show an obviously wider distribution than normal liver (open circle) and cirrhosis samples (blue circle). (**C**) The MDS plot of ***St*s **data set. Liver and spleen samples are labeled with opened and filled circle respectively.

We collected six independent microarray data sets from two public archives (see *Methods*) to demonstrate the heterogeneity of tumor samples. We measured sample heterogeneity by calculating the average Pearson dissimilarity distance between paired samples within a sample type (Figure 2A). We found that most tumor samples (gray bars), including hepatocellular carcinoma (HCC), lung and colon tumors, were more heterogeneous than normal and precancerous tissues. Even for the most initial stage of HCC (very early HCC, # in Figure 2A), the sample heterogeneity is significantly higher than that of normal livers (*p *= 1.73E-5) or pre-cancer samples (*p *= 3.38E-9). We further used multidimensional scaling (MDS), a dimensional reduction algorithm [10], to visualize the heterogeneity of tumor samples. The distance in MDS plot represents the degree of Pearson dissimilarity among samples. When transcriptomes of samples are similar (i.e., homogeneous), the samples will be closely clustered. In HCC^2 ^data set (Figure 2A), four types of non-tumor samples (normal liver, cirrhosis, low-grade and high-grade dysplasia) are distinctly distributed in the MDS plot (see Additional file 1), and those samples are closely clustered according to their clinical type. However, such clustering was not seen in HCC samples (see Additional file 1).

### The limitation of gene-phenotype approaches on tumor samples

**Figure 3:**
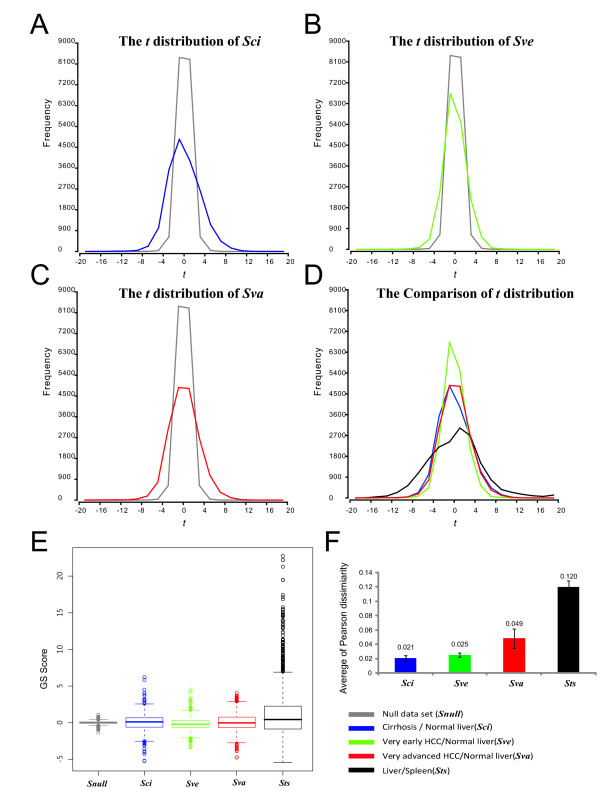
**Comparisons of homogeneous and heterogeneous data sets with their corresponding null data sets**. (**A**-**C**) The *t *distributions for ***Sci***, ***Sve ***and ***Sva ***data sets are compared with the corresponding null data set (gray line). (**D**) The *t *distributions of four test data sets are compared in the overlapped histogram. (**E**) The GS score (averaged *t *values) of 1644 GSs are compared in the box plot. (**F**) The transcriptomic changes between sample types were measured by average Pearson dissimilarity distance. The average distances of four test data sets are shown in bar-chart.

The biological interpretations of microarray experiments are often performed by gene- or gene set-based approaches according to the gene-phenotype correlations. However, such correlation is barely detectable in heterogeneous genomics data sets (such as tumor transcriptomes) so that the detection power of either approach is decreased. To illustrate the influence of sample heterogeneity on data analysis, we selected data sets from three different liver pathological stages (***Sci***: cirrhosis, ***Sve***: very early HCCs and ***Sva***: very advanced HCCs in HCC^2 ^experiment) to present the different levels of transcriptomic changes and heterogeneity. Six samples of each stage were compared with six normal liver samples to identify changes in gene expression. As shown in Figure 3F, the transcriptomic changes increase along with the HCC progression. ***Sva ***have the greatest changes from normal liver (average Pearson dissimilarity: 0.049 ± 0.013), indicating many genes are de-regulated. The other early stages have relatively small changes and the change levels of ***Sci ***and ***Sve ***are similar (***Sci***: 0.021 ± 0.004 and ***Sve***: 0.025 ± 0.003, Figure 3F).

The transcriptomes of cirrhosis samples (§ in Figure 2A) are homogeneous and their transcriptome profiles (blue circle) as well as that of normal livers (open circle) are closely self-clustered in MDS plot (Figure 2B). Both very early HCC (# in Figure 2A) and very advanced HCC samples (‡ in Figure 2A) are heterogeneous and their transcriptome profiles are dispersed (very early HCC: green circle, very advanced HCC: red circle; Figure 2B). We also added an extreme case, 4 normal liver and 4 normal spleen samples in the analysis. The gene expression patterns of normal tissues are tightly controlled by tissue-specific regulation. The high homogeneity is shown in Figure 2C and ¤ in Figure 2A. A huge transcriptome difference (0.120 ± 0.009, ***Sts***: Tissue comparison in Figure 3F) can be detected between the two tissue types.

To estimate background variations for each data set, we compared the *t*-distributions of these four data sets (***Sci, Sve, Sva ***and ***Sts***) with their corresponding null data sets (***Snull***), which have the same sample number but their gene signals were randomly sampled from normal distribution (μ = 0, σ^2 ^= 1). In such comparison, heavier tail distribution indicates more genes have significant gene-phenotype correlations, i.e., data sets with heavy tails are more readily being analyzed by conventional *t *value-based methods. As an extreme case, ***Sts ***showed the most heavy tail distributions (black line in Figure 3D) among all tested data sets. We then compared two data sets with similar transcriptomic change level (***Sci ***and ***Sve***, Figure 3F). The tail distributions of ***Sci ***(blue line, Figure 3A) are heavier than those of ***Sve ***(green line, Figure 3B). Even in ***Sva ***(red line, Figure 3C), which contains over 2-fold dissimilarity than ***Sci ***(Figure 3F), the tail distributions (red line, Figure 3D) were still smaller than those of ***Sci ***(blue line, Figure 3D). The variation of *t *value is less informative in heterogeneous data sets. Classical approaches are therefore more applicable to homogenous data sets, but not heterogeneous ones (such as clinical tumor samples).

**Figure 4:**
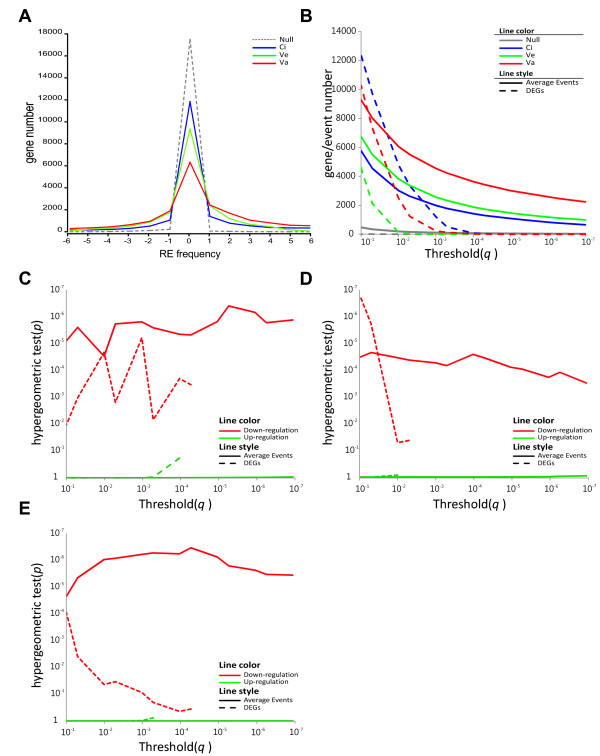
**Threshold insensitivity and robustness of RE-based analysis**. (**A**) The RE frequency distributions for three liver data sets (blue: ***Sci***, green: ***Sve ***and red: ***Sva***) and corresponding null data set (gray dash line: ***Snull***). The x-axis represents the gene RE frequency (up minus down) and the y-axis represents the gene numbers. (**B**) The comparison of threshold sensitivity for DEG (dashed line) and average RE (solid line) detections. Total regulations, including up and down direction, were counted by various detection thresholds (*q*-value, x-axis). The y-axis represents the number of filtered DEGs or REs. (**C-E**) The detection robustness of enrichment test for GS "lipid metabolic process" (GO:00006629) were compared between DEGs and average RE. The down-regulation and up-regulation are separately shown in red and green color, respectively. The y-axis represents the significance level of the GS across various detection thresholds of DEG or average RE.

In IGA, the function annotation enrichment analysis is based on the most significant de-regulated genes (Figure 1). These genes are selected from both ends of the *t*-statistic distribution, which are the most informative regions. Hence, IGA is expected to be a sensitive method for heterogeneous samples. However, de-regulated genes can barely be identified from heterogeneous and mild-changed samples, such as ***Sve ***(Figure 3B and 4B). The selection of thresholds presents researchers with the dilemma of obtaining accurate interpretations but only few significant DEGs or obtaining more DEGs by reducing selection threshold (i.e., by reducing statistic powers) to expand the scope of analysis.

In gene set-based analysis (GSA), the GS score, which is summarized from gene-phenotype correlations of all members in a gene set, is used to estimate functional changes (Figure 1). Because GSA bypasses the threshold selection step, it can avoid the dilemma mentioned above. However, we wondered if such algorithms, based on the gene-phenotype correlation, also decrease their detection power in heterogeneous samples. We calculated the average *t*-statistic for each gene set (GS) to get GS scores. The increasing or decreasing of GS score represents the degree of gene set correlation with phenotype. We compared scores distribution of all data sets in Figure 3E. ***Sts ***shows the most significant GS score variation (-5.5~22.7), which represents the obvious function difference between liver and spleen. The score variations of ***Sci***, ***Sve ***and ***Sva ***are much smaller than ***Sts ***since they have similar tissue background when compared with reference samples, the normal liver. Although ***Sci ***has the smallest transcriptomic changes (Figure 3F), ***Sci ***shows a more significant GS score variation (-5.22~-6.20) than the other data sets do (***Sve***: -3.35~4.52, ***Sva***: -4.75~4.09). The result implies that the heterogeneity of tumor samples reduces the detection power of GSA too.

### Threshold insensitivity and robustness of eGSA

Recognizing the limitations of current gene- or gene set-based methods on heterogeneous samples, we alternatively analyzed transcriptomic changes by using regulatory events. Regulatory event is defined as a gene signal change of a test sample compared with a reference sample pool (see *Materials and Methods*). The distribution of regulatory event frequency in all data sets is clearly distinguishable from null data sets (Figure 4A) and the gradual increasing of tail distributions correlates with their transcriptomic change levels (Figure 3F). This result implies regulatory event is a better approach for analyzing heterogeneous samples. Based on this observation, we designed a novel regulatory event-based strategy, which we called the regulatory **e**vent-based **G**ene **S**et **A**nalysis (eGSA), for the exploration of significant gene sets and compensate for the insufficiency of traditional method in heterogeneous samples.

One might argue that the regulatory event determination is still under an arbitrary cut-off threshold and its results also suffer from the same dilemma of threshold selection as IGA does. This argument can be partially answered by the insensitivity of regulatory event detection in different threshold values (see Figure 4B). The number of differentially expressed genes (DEGs) is sensitive to the threshold selection and is dramatically decreased when a stringent threshold is applied. Under a strict threshold (*q*-value < 1.0E-3), 1204 genes could be detected in homogenous data set (***Sci***, Figure 4B). However, only a few differentially expressed genes could be detected in heterogeneous data sets (***Sve***: 1 and ***Sva***: 229, Figure 4B). In contrast, even under the most stringent threshold in our test (*q*-value < 1.0E-7), thousands of regulatory events could be detected robustly. Moreover, the event numbers of those data sets also correlated with the level of transcriptomic changes (Figure 3F).

The threshold insensitivity of regulatory event detection also reflects the robustness of eGSA. We selected the lipid metabolic process (GO:0006629) category in the GO databases as an example because both liver cirrhosis and carcinogenesis cause the defective lipid metabolism [11,12]. Obvious down-regulation of this functional category is expected in all ***Sci***, ***Sve ***and ***Sva ***data sets (red line, Figure 4C–E). In IGA, hypergeometric test *p*-values increase heavily toward the background values of the opposite regulation when higher threshold stringency was used in heterogeneous data set (dash line, Figure 4D–E). IGA even loses the detection power due to no annotated de-regulated genes (dash line, Figure 4C–E). Although the homogeneous data set, ***Sci***, showed higher tolerance to stringent thresholds, the *p*-values appeared unstable when different thresholds were selected (red dash line, Figure 4C). Nevertheless, in eGSA the *p*-values are more stable in both homogeneous and heterogeneous data set (red solid line in Figure 4C–E) and could be distinguished from the background values in all tested threshold values. Conclusively, measuring regulatory events is insensitive to threshold selection and can be performed more robustly in hypergeometric test.

### Precise and broad biological interpretation of transcriptomechanges by eGSA

**Figure 5:**
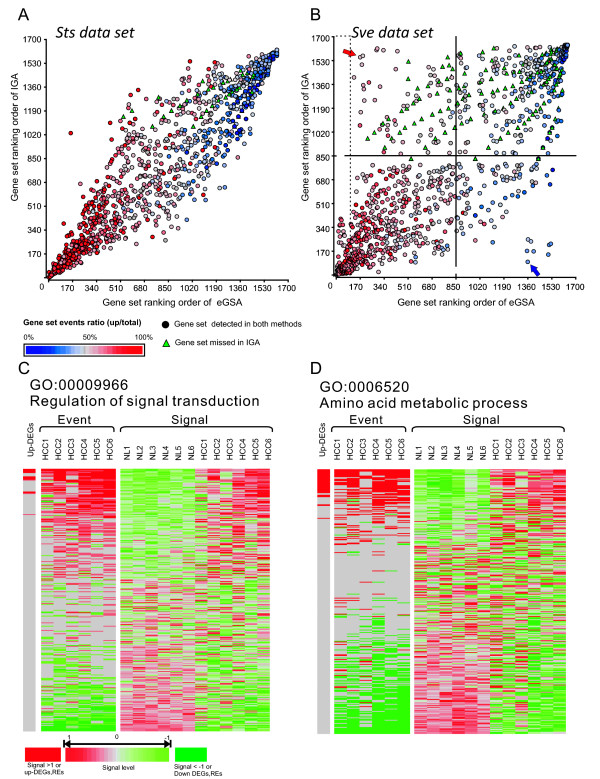
**Correlations between eGSA and IGA**. (**A**) and (**B**), Scatter plots of GS significance ordered by eGSA (y-axis) and IGA (x-axis). Each circle represents one GS, and green triangles represent missed GSs in IGA. The color represents the percentage of up-regulated REs in a GS (RE_up in GS_/RE_total in GS_). Most GSs in ***Sts ***are correlated and aligned on the diagonal line. In ***Sve***, the high- and low-rank GSs are also correlated but many GSs appear inconsistent (in 2^nd ^and 4^th ^quadrant). Their gene changes of two extremely inconsistent GSs, which are indicated by red and blue arrows, are further shown in (C) and (D). In these heatmaps, each row represents one gene in a GS while each column represents a tissue sample. The first column is the up-DEGs detected by IGA (shown in red). The event panel is REs detected by eGSA (red: up-REs; green: down-REs), while the signal panel is the standardized (mean = 0, SD = 0.5) microarray signals of all samples. The signal level is presented by the color scale (red: up-regulation; green: down).

We further compared the accuracy and analysis scope between eGSA and IGA. Using 1634 gene sets defined by GO terms, we calculated the significance levels of gene sets based on the up-regulated genes or regulatory events. To make these two results comparable, we ranked gene sets by their *p*-values and then present their ranking orders in a scatter plot (Figure 5A and 5B). For every gene set, we also calculated the ratio of up-regulated regulatory events over the total regulatory events within the gene set to indicate their regulatory directions.

In ***Sts***, the most distinctive data set (Figure 3F), the ranking orders of gene set are highly correlated between eGSA and IGA (Figure 5A), suggesting that eGSA has comparable detection power to that of IGA in homogeneous samples. This is due to the fact that most of the consistent gene regulations in ***Sts ***can be detected by both strategies. In ***Sve***, the most indistinctive data set (Figure 3F), the top- and bottom-ranked gene sets of IGA and eGSA are correlated and are aligned in the diagonal corners (Figure 5B). This is because these gene sets represent the most consistent changes. However, eGSA detected more up-regulatory gene sets that were under-estimated (red nodes in the 2nd quadrant) or missed (green triangle) by IGA. This data demonstrates that eGSA has a broader scope of analysis than IGA. Moreover, several down-regulated GSs (blue nodes in the 4th quadrant) are misidentified as up-regulated GSs by IGA (Figure 5B).

We selected two extremely contradictory gene sets to demonstrate the different preferences between these two methods. The first gene set, the regulation of signal transduction (GO:0009966; indicated by a red arrow in Figure 5B), is highly-ranked in eGSA (170^th^) but not in IGA (1554^th^). The second gene set, the amino acid metabolic process (GO:0006520; blue arrow in Figure 5B), is highly ranked in IGA (94^th^) but not in eGSA (1359^th^). The details of both gene sets, including their differentially expressed genes, regulatory events and signal values, are shown in Figure 5C and 5D. In the first gene set, regulatory event-based analysis shows that 20% of genes (total regulatory events/(total genes × total test samples)) are up-regulated, which is in agreement with the observation in sample signal values. However, only 4% up-regulatory de-regulated genes can be detected due to the lack of consistency in gene expression level (Figure 5C). This also suggests that IGA may sometimes under-estimate the significance of a gene set. In the second GS, IGA detects a certain number of consistently up-regulatory de-regulated genes and conclude this gene set as a significantly up-regulated one (*p *= 7.99E-05). But this doesn't perfectly present the fact that there are actually more down-regulatory (24%) than up-regulatory (15%) expression changes in this gene set (Figure 5D). We conclude that for heterogeneous samples, since eGSA takes into account all REs in a gene set, this novel strategy can overcome certain limitations of IGA.

### Functional patterns of very early HCC

To demonstrate the advantage of eGSA, we applied eGSA on the discovery of biological functions associated with liver cancer tumorigenesis. Hepatocellular carcinoma (HCC) is characterized as an obvious multistage process, just like many other types of human tumors. Understanding the de-regulated biological functions in early stage HCC will help to reveal the initial processes of hepatocarcinogenesis. The insights of early HCC functional changes and regulatory mechanisms are far from clear due to the lack of common oncogenes and tumor suppressors. One of the major difficulties in identifying common regulatory mechanisms is the genetic heterogeneity of HCCs [13,14]. To overcome this heterogeneity issue, we applied eGSA on the analysis of the initial functional changes of HCV-induced HCCs (HCC^2^) [15].

**Figure 6:**
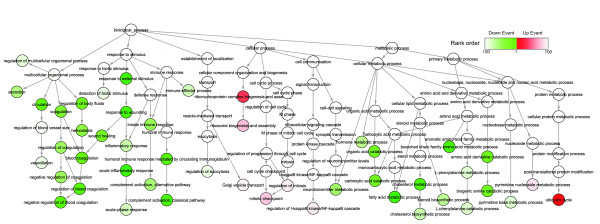
**Major functional changes in very early HCCs**. The top ranked GSs (the dash box in Figure 5D) are organized into an acyclic network according to their GO term relationships. Each node represents one GS, and node color represents its ranking order by eGSA (up-REs in red while down-ones green). The GO term of a GS is labeled on the node. Several linking nodes (white) are added to cluster nodes into function-related branches.

In the original paper of HCC^2 ^only 104 differentially expressed genes were identified when dysplasia samples (n = 17) were compared to early HCCs (n = 18) [15]. The authors interpreted the biological function of early HCC based on gene functional classification. As a result, only a few gene sets were analyzed and their statistical significance had not been estimated yet. To provide a more comprehensive view on the initial processes of hepatocarcinogenesis, we applied eGSA on the earliest stage of HCCs (very early HCC in Figure 2A, n = 6). Owing to the insensitivity of eGSA to transcriptomic heterogeneity, we can now identify more altered biological functions with statistical significance in very early HCC. To avoid potential errors from name-space issues [2], we carefully removed the gene sets containing high percentage of duplicative genes (>10%). The filtered gene sets were functionally clustered into branches of an acyclic network according to their GO term relationships (Figure 6). The GS ranking orders are presented in a color scale (red: up-regulated; green: down-regulated). The top 100 up- or down-regulated gene sets are listed in Additional file 2.

In the eGSA results, the major functional changes are basically consistent with the original observations and previous descriptions on HCC [11,15-17]. Moreover, eGSA provides new insights into the initial processes of HCC. Gene sets related to major hepatic metabolisms, including cholesterol, steroid, hormone and amino acid metabolisms, are significantly down-regulated (see section B of Additional file 2). The down-regulation of these biological functions may be due to the failure of normal hepatic functions. On the contrary, nucleoside and protein metabolisms required for rapid cell growth are significantly up-regulated. These include pyrimidine nucleoside metabolism (*p *value is 2.3E-5, ranking order is 89^th^), ubiquitin-mediated protein degradation (4.76E-5, 37^th^), tRNA aminoacrylation (3E-3, 98^th^) and ribosome biogenesis and assembly (9.52E-4, 76^th^) (see section A of Additional file 2).

The down-regulation of lipid metabolisms has been frequently reported in liver diseases [11,12]. In the initial stage, fatty acid metabolism (2.6E-5, 22^th^), steroid biosynthesis (6.5E-3,77^th^) and sterol related metabolisms, e.g. hormone metabolism (6.4E-3,76^th^), are all down regulated since several common enzymes are shared within these metabolic processes (see B section in Additional file 2). Although lacking a consistent conclusion, several studies did report that a sterol hormone, androgen, was associated with HCC development: high level serum androgen was found as risk factor for HCC [18], and androgen receptor has also been reported to promote cell growth and anti-apoptosis in hepatic cell line model [19]. Hence, the linkages between the down-regulations of sterol metabolisms and the abnormal hormone metabolisms, and their effects on HCC initiation could be interesting and worthy of further study.

Escaping and surviving from attacks by the immune system are two other significant functional changes in HCC initiation. Certain immune functions, such as the inflammatory response (8.0E-3, 81^th^), innate immune response (3.5E-3, 64^th^) and immune effector process (9.2E-3, 86^th^), are down-regulated while the survival pathway, the regulations of IκkB/NFκB cascade [20,21] (2.6E-3, 93^th^), is up-regulated (Additional file 2). These observations are supported by many studies. For example, the number of different types of inflammatory cells are decreased in HCC tissue sections [22]; the suppression of T cell-mediated immune responses is found in HCC patients and is associated with poor prognosis [23,24]. To understand the regulatory mechanisms, we analyzed the biological pathways involved in those immune-related gene sets. Genes with high regulatory event frequency (up + down > 0.8) were annotated with their involved KEGG pathways [25], and the most annotated pathways are listed in Additional file 3. Several biological signaling pathways are highlighted, including the Toll-like receptor, JAK-STAT, MAPK and T cell receptor signaling pathway. Besides, a number of immune-related processes are also highlighted, such as the cell adhesion molecules, cytokine-cytokine receptors, antigen-processing and presentation, and NK cell mediated cytotoxicity (see Additional file 3).

**Figure 7:**
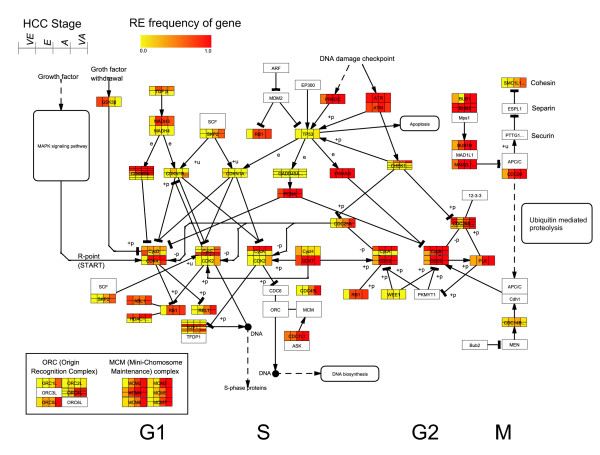
**The gene RE frequencies in the cell cycle pathway**. The gene up-RE frequencies at each HCC stage are mapped as a grid on the cell cycle pathway (KEGG: hsa04110) by using a public software PathwayExplorer [9]. In a grid, columns represent very early (VE), early (E), advance (A) and very advance (VA) HCCs, respectively. Rows represent genes annotated with the identical function in the pathway. The representative gene names are labeled in the center of the grids.

It is not surprising that cell cycle is up-regulated at the initial stage of HCC. However, in an advanced comparison of cell cycle phases, we identified that M phase (4E-3, 111^th^) is the most significant phase over G1 (2.4E-1, 554^th^) and S (2.5E-2, 180^th^). M phase related gene sets, such as regulation of mitosis (1E-2, 94^th^) and mitotic cell cycle checkpoint (1E-3, 79^th^), are both significantly up-regulated (Figure 6 and section A of Additional file 2). To further illustrate the early changes of M phase regulation, we mapped the up-regulated regulatory event frequencies of four stages (very early, early, advance and very advance HCC stages) on a KEGG cell cycle pathway (hsa04110). As shown in Figure 7, most G2/M key regulators (CCNA2, CCNB and CDK1) are up-regulated at the first stage (also see Addtional file 2, the dashed box in panel A). These genes appear earlier than many G1/S key regulators (RB, RBL1, CDK2, CDK4 and CDK6) (Figure 7). The early up-regulation of G2/M phase can also be supported by the appearance of the corresponding down-stream genes. For example, the regulators of mitosis and chromosome segregation (BUB1, BUB1B, BUB3, MAD2L1 and CDC20) were frequently up-regulated at the first stage, while the regulators of DNA replication (ORC and MCM complex) are up-regulated only at the later stages (Figure 7). In another sample set (HCC^1^), although lacking in stage information, we still found that these G1/M regulators were more frequently and synchronously up-regulated (see panel B of Additional file 4). On the contrary, G1/S regulators, which are accompanied by DNA synthesis, are seldom found (panel B of Additional file 4). Our findings are consistent with the previous protein and kinase activity studies. The enhancement of CCNA/CDK1 protein expression and kinase activity are observed in early HCC stage and non-tumorous cirrhosis tissues, but not in normal livers. G1/S regulators, CCND1, CCNE1 and CDK4, are higher in poorly differentiated HCC and advanced HCCs [26].

CCNA2 is considered as an important factor leading to cancer because of its dual roles in both S and M phase [27,28]. The increased expressions of CCNA2 mRNA and protein were observed in many clinical HCC studies [27,29,30]. However, in cell model experiments, over-expression of CCNA2 alone could not promote cell cycle but delayed metaphase/anaphase onset [31]. These findings suggest that the high expression of CCNA2 might simply reflect high tumor proliferation rate rather than promote tumorigenesis [27].

In our study, CCNA2 appears as a most frequent and earliest up-regulated cyclin at the initial stage. This strongly suggests that CCNA2 might play a crucial role in HCC initiation. Accompanying CCNA2, several mitosis-related regulators, such as CDC2, CCNB1/2 and CDC20, are synchronically up-regulated (Figure 7), indicating that all these mitosis genes are also required for HCC initiation. However, previous individual gene studies did not test the gene combination (see Additional file 4). Besides, an interesting supportive mechanism, the up-regulation of ubiquitin mediated proteolysis activity (4.76E-05, 37^th^), could also potentially contribute to the rapid turn-over of cyclin and other mitosis components, and then help to complete the cell cycle (see section A of Additional file 2).

## Conclusion

The common transcriptomic heterogeneity of tumor reduces the detection power of gene-list or gene-set analysis to identify functional patterns of transcriptome profiles. We developed eGSA, a novel method based on regulatory events, to overcome such limitations. eGSA is insensitive to threshold bias and can provide more robust and precise results than IGA. These properties make eGSA an excellent approach to analyze complex tumor transcriptome profiles.

eGSA overlooks the highly heterogeneous regulatory mechanisms and directly targets the question "what happened on cellular functions?". Once functional issues are addressed, the regulatory event frequency can be applied to highlight potential regulatory routines in the gene network and to present their prevalent regulatory mechanisms. The identification of these regulatory routines will greatly accelerate the development of pharmaceutical targeting strategies and personalized therapy.

Along with this study, we also noticed a few limitations of eGSA. First, the determination of regulatory events is heavily dependent on a stable and highly correlated reference pool. This requirement cannot always be met in many cases, such as the comparison between two heterogeneous sample types. Second, the biological interpretation of eGSA relies heavily on the definition and composition of a gene set. Several unsolved problems, such as name-space issues, imprecise or incorrect annotations, will interfere with eGSA results. It still lacks an optimization process to avoid these problems so far. Finally, like most gene set-based analysis, eGSA counts the contribution of each gene in biological regulations equally. This equality is not always true in a cell because certain genes are more crucial in changing the whole cellular function than others. Hence, an advanced weighting algorithm of gene set definition remains to be developed for more precise biological function interpretation.

## Methods

### Data collections and preprocessing

Six independent data sets (Normal, HCC^1^, HCC^2^, Tumor^1^, Tumor^2^, Tumor^3^), including one normal tissue data set (GSE3526), two HCC data sets (E-TABM-36 and GSE6764), and data sets for other three tumor types: thyroid cancer (GSE3678), colon cancer(GSE4107) and lung cancer (GSE7670), were downloaded from two public archives (NCBI GEO [32] and ArrayExpress [33]). The sample accession numbers and tissue types are listed in Additional file 5. All data were implemented in Affymetrix U133A and U133 Plus 2.0 platform, and the raw data (.CEL) were normalized by RMA algorithm using statistic software Partek^® ^Genomics Suite™ (Partek Inc., St. Louis, MO, USA). Affymetrix probe set IDs were converted to UniProt IDs before mapping onto the GO database. The expression level of each UniProt ID (gene) is the average of the corresponding probe set signals. In total, 26300 genes were summarized from 54675 probe sets of U133 plus 2.0 platform and 18033 genes were annotated in GO.

### Gene set (GS) definition

GS is comprised of genes with the same GO term. By using the biological process category of GO, there are 4903 GSs encapsulated from the Affymetrix U133 Plus 2.0 platform. After excluding the GSs containing less than 10 genes, 1643 GSs were used for GS-based analysis. The GS mapping, gene ID converting and summarization were processed by Microsoft Access 2007 and VBA.

### The measurement of sample heterogeneity

We measured the Pearson dissimilarity distance (defined as [1 - r]/2, where r is the Pearson correlation) to present the transcriptome difference between two samples. The heterogeneity of a sample type was presented by the mean of distances between paired samples. The transcriptomic change between two sample types was presented by the mean of all pairwise distances between members of the two groups concerned. Multidimensional scaling plot (MDS) was used to illustrate the distances among samples in a data set [10]. The calculation of Pearson dissimilarity distance and MDS plotting were performed using the statistical software Partek Genomic Suit.

### The measurement of gene-phenotype correlation

In homogeneous data sets, the gene-phenotype correlations were measured by the Student's *t*-test. Alternatively, Welch's *t *test was applied for the heterogeneous data set due to the possible unequal variance observed in the two samples types. The *t-*statistic of each gene was applied for GSA scoring (average *t*-statistics) and *t*-distribution plot. For detecting differentially expressed genes, the multiple-test correction of *p*-value was performed by positive false discovery rate (*q*-value) [34] and genes were filtered by a given *q*-value threshold. The *t-*statistic of genes and positive false discovery rate (*q*-value) were calculated by the Partek Genomic Suit.

### Signal-to-event conversion

In a data set, samples were classified into test and reference groups. The statistical significance of gene signal (x) in a test sample was estimated by the cumulative probability distribution of the normal distribution. The signal mean (*μ*) and standard deviation (*σ*) of reference sample pool were calculated and applied to cumulative probability distribution function in Microsoft Excel, NORMDIST(x, *μ*, *σ*, True). The multiple-test correction of *p*-values was performed by *q*-value. The significant signal change, as an expression regulatory event (RE), was determined by a given significance cut-off. According to the regulated direction, RE was defined as up-regulatory event (up-RE) or down-regulatory event (down-RE).

### Event-based gene set analysis (eGSA)

eGSA contains two processes to estimate the significance level of a gene set (GS): (1) GS scoring and (2) Gene set statistic (Figure 1).

#### (1) GS scoring

In an event matrix, up-regulatory event (RE) frequency and down-RE frequency were calculated respectively as:

*Gene RE frequency *= Σ*RE*_*j*_/*j*,

where *j *is the sample number of a given test sample type. A GS score (*k*) is the sum of gene RE frequency in a GS and calculated as:

*k *= Σ*RE*_*ij*_/*j*,

where *i *is the gene number of a GS.

#### (2) Gene set statistic

The probability of observing at least *k *from a particular GS is tested by hypergeometric distribution.

$$P=1-\sum_{i=0}^{k-1}\frac{\left(\begin{array}{c} m\\ i \end{array}\right)\left(\begin{array}{c} N-m\\ n-i \end{array}\right)}{\left(\begin{array}{c} N\\ n \end{array}\right)},$$

where *m *is the total gene number in a GS, *N *is the total gene number in a microarray platform, and *n *is the average REs in a sample type. The calculations of hypergeometric test were performed by R packages .

### Simulated null data set

We generated a null data set to mimic random variation of a testing data set. The null data set has the same sample number and gene number as the test data set but its signal values were randomly sampled from normal distribution. The simulated data set was generated by using a standard function, NORMSINV(RAND), in Microsoft Excel 2007.

## Abbreviations

DEG: differentially expressed gene; IGA: individual gene analysis; GSA: gene-set analysis; GS: gene set; eGSA: event-based gene set analysis; RE: expression regulatory event; GO: Gene Ontology.

## Authors' contributions

CYT conceived the study is valuable for cancer research. CYT, CHJ, and HWW designed the analysis approaches. CYT collected microarray data sets. CYT and CHJ carried out the implementation of data analysis, and wrote the manuscript. HWW, MTH, and CHL provided biological guidance during the analysis process. All authors read and approved the final manuscript.

## Supplementary Material

Additional file 1 **Relationships of HCV-induced HCCs.** The inter-sample dissimilarities of HCCs are presented in a MDS plot. Each node represents one sample and colors of the nodes represent their clinical stages as indicated. Click here for file

Additional file 2 **The major functional changes of very early HCCs.** The top 100 ranked GSs by up-(**A**) or down-RE (**B**) eGSA. The corresponding results from IGA are also listed. The "Hits" panel represents the average RE number and DEG number in a gene set. The "Significance" panel shows the *p*-value of hypergeometric test. The Err% panel represents the percentage of duplicative genes, which are defined as several genes measured by a single probe set of the Affymetrix chips. Total error% indicates the percentage of duplicative gene in a GS and the Hits error% indicates duplicative DEG in a GS. The GS information is listed in the last three columns, included GO id, gene number and GO term. Click here for file

Additional file 3 **Cross-analysis of high regulatory event frequency genes between gene sets and KEGG pathway.** The overlapped gene numbers are shown in the "Hits" column and the pathways they are involved in are listed. Click here for file

Additional file 4 **The event tables of cell cycle regulators in two independent microarray experiments.** 35 central regulators are listed in rows and are grouped according to their roles in cell cycle pathways, including the G2/M, M, G1/S, RB/E2F, and S phase (see also Figure 7) (A) In HCC^2 ^data set, 63 samples are aligned in the columns according to progressive HCC stages. Each cell represents the detection of RE (up-RE: red, down-RE: green, no change: gray). In very early HCCs (dash box), the RE frequency of G2/M and M phase are higher than that of RB/E2F, G1/S and S phases. (B) In another experiment (HCC^1^), G2/M regulators are also the most frequently up-regulated genes. Click here for file

Additional file 5 **Data set information.** * obtained from NCBI GEO [32]. ^# ^obtained from ArrayExpress [33]. Data set reference: HCC^1 ^[15], HCC^2 ^[17], Tumor^1^[35], Tumor^3 ^[36] Click here for file
