# Supplementary figures and images for: A novel regulatory event-based gene set analysis method for exploring global functional changes in heterogeneous genomic data sets
Source: BMC Genomics. 2009 Jan 16;10:26. doi: 10.1186/1471-2164-10-26 (PMC2637897; doi:10.1186/1471-2164-10-26)

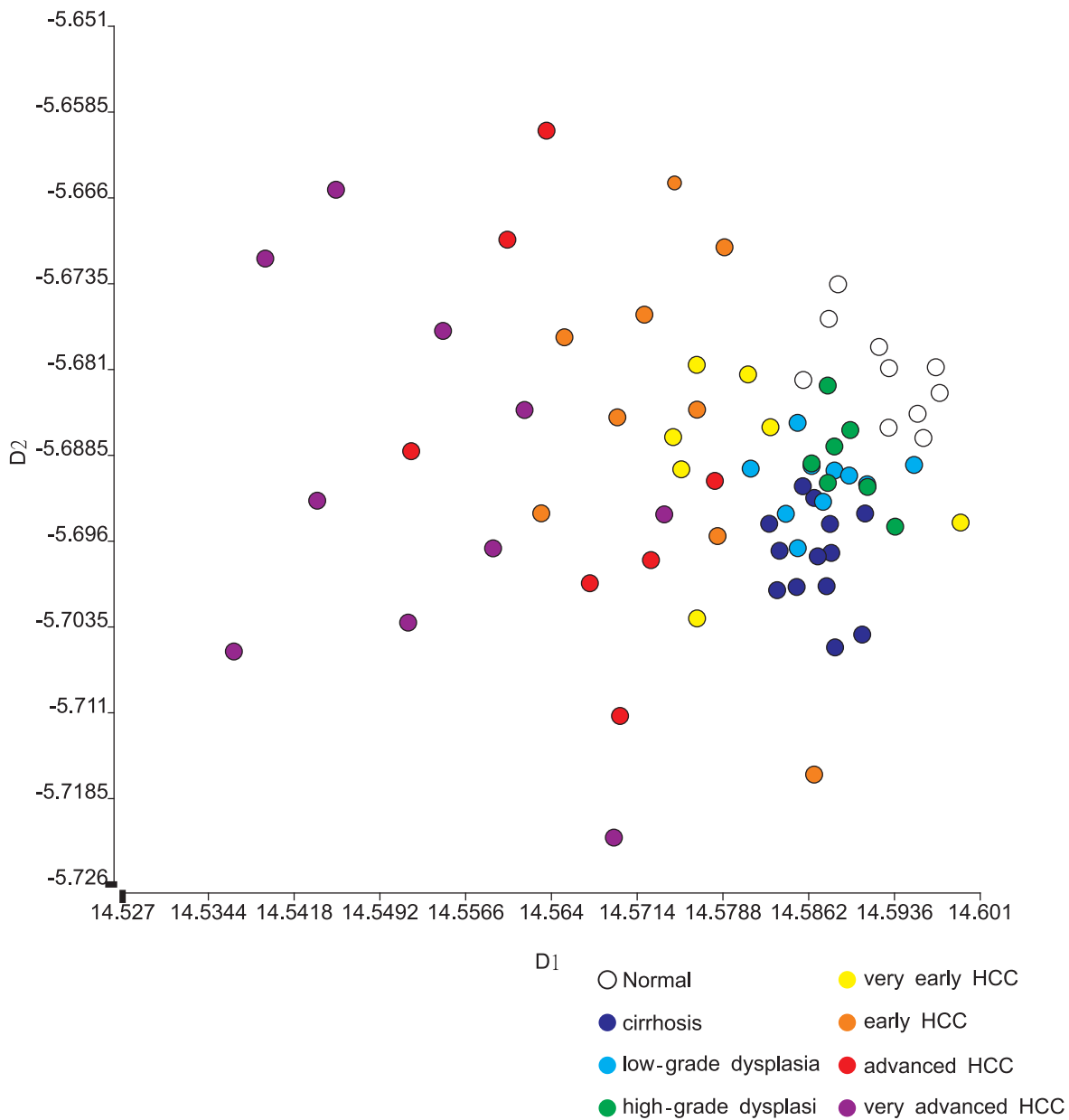

Supplement: Additional file 1 — Relationships of HCV-induced HCCs. The inter-sample dissimilarities of HCCs are presented in a MDS plot. Each node represents one sample and colors of the nodes represent their clinical stages as indicated. [file 1471-2164-10-26-S1.pdf]

(A) HCC<sup>2</sup> (GSE6764)

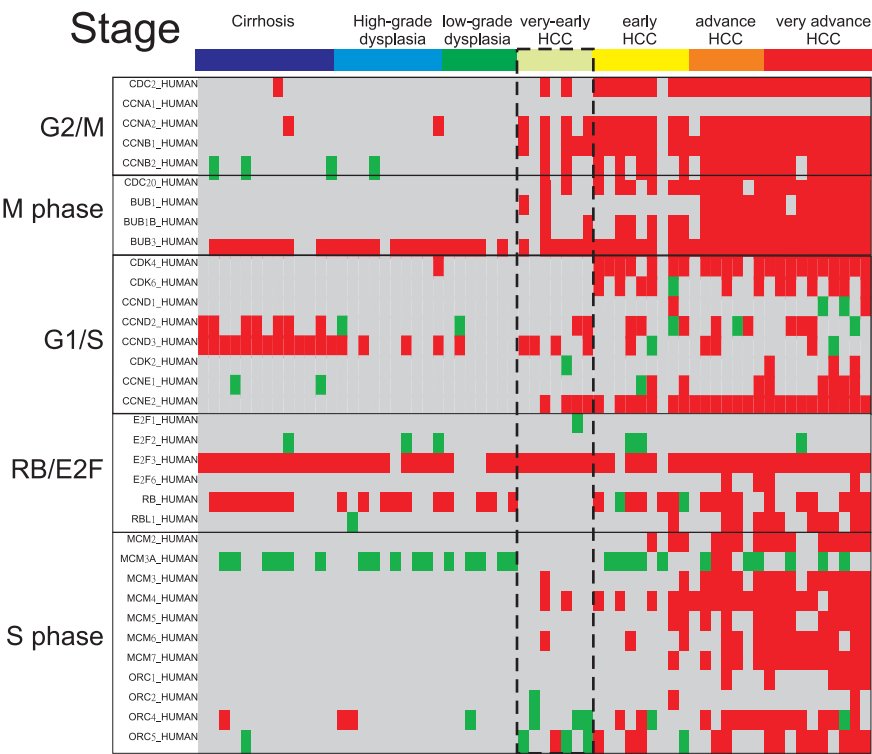

(B) HCC<sup>1</sup> (E-TABM-36)

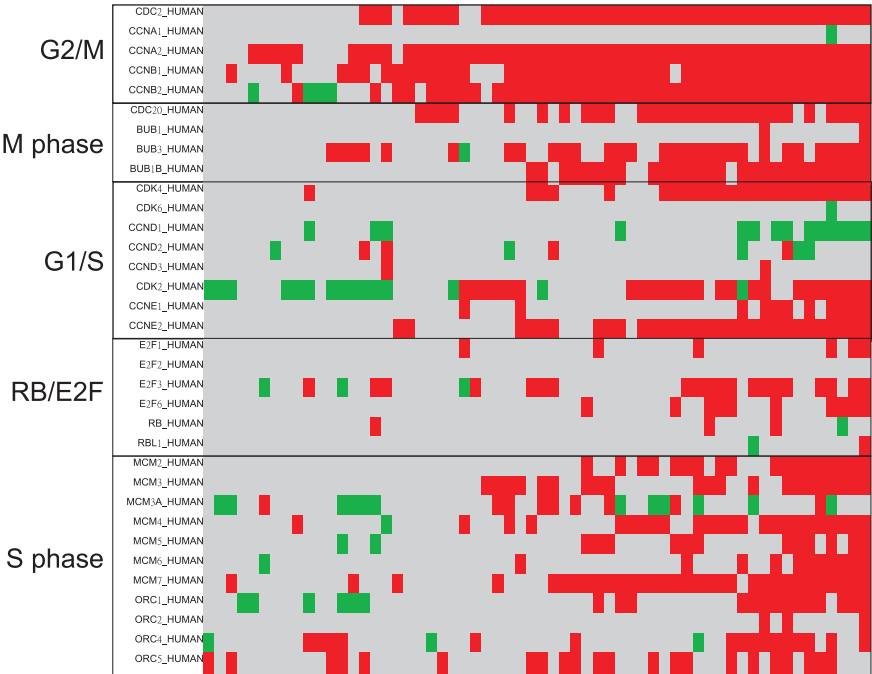

Supplement: Additional file 4 — The event tables of cell cycle regulators in two independent microarray experiments. 35 central regulators are listed in rows and are grouped according to their roles in cell cycle pathways, including the G2/M, M, G1/S, RB/E2F, and S phase (see also Figure 7) (A) In HCC2 data set, 63 samples are aligned in the columns according to progressive HCC stages. Each cell represents the detection of RE (up-RE: red, down-RE: green, no change: gray). In very early HCCs (dash box), the RE frequency of G2/M and M phase are higher than that of RB/E2F, G1/S and S phases. (B) In another experiment (HCC1), G2/M regulators are also the most frequently up-regulated genes. [file 1471-2164-10-26-S4.pdf]
