# Supplementary material for: A novel regulatory event-based gene set analysis method for exploring global functional changes in heterogeneous genomic data sets
Source: BMC Genomics. 2009 Jan 16;10:26. doi: 10.1186/1471-2164-10-26 (PMC2637897; doi:10.1186/1471-2164-10-26)
Supplement: Additional file 2 — The major functional changes of very early HCCs. The top 100 ranked GSs by up-(A) or down-RE (B) eGSA. The corresponding results from IGA are also listed. The "Hits" panel represents the average RE number and DEG number in a gene set. The "Significance" panel shows the p-value of hypergeometric test. The Err% panel represents the percentage of duplicative genes, which are defined as several genes measured by a single probe set of the Affymetrix chips. Total error% indicates the percentage of duplicative gene in a GS and the Hits error% indicates duplicative DEG in a GS. The GS information is listed in the last three columns, included GO id, gene number and GO term. [file 1471-2164-10-26-S2.pdf]

A

Rank		Hits		Signifincance		Err%		Gene set info		
eGSA	IGA	eGSA	IGA	eGSA	IGA	Total	Hits	GO id	Gene #	Title
1	2	68	80	1.75E-36	7.23E-91	78.9%	87.5%	GO:0002474	90	antigen processing and presentation of peptide antigen via MHC class I
2	3	69	81	4.63E-35	1.53E-88	74.7%	86.4%	GO:0048002	95	antigen processing and presentation of peptide antigen
3	4	106	109	1.86E-21	3.22E-67	68.2%	84.4%	GO:0019882	261	antigen processing and presentation
4	1	2963	1037	1.11E-16	0	25.5%	31.1%	GO:0008150	18033	biological_process
5	7	547	241	1.22E-07	1.54E-12	12.1%	16.2%	GO:0016043	2758	cellular component organization and biogenesis
6	34	198	89	1.86E-07	2.79E-08	11.3%	11.2%	GO:0033036	865	macromolecule localization
7	61	740	281	1.95E-07	3.55E-06	23.1%	24.2%	GO:0044267	3867	cellular protein metabolic process
8	60	778	296	3.20E-07	2.56E-06	22.5%	23.6%	GO:0019538	4092	protein metabolic process
9	30	188	86	3.63E-07	2.08E-08	11.8%	11.6%	GO:0008104	821	protein localization
10	33	181	83	3.64E-07	2.38E-08	12.2%	10.8%	GO:0045184	785	establishment of protein localization
11	5	196	133	4.19E-07	9.26E-27	28.9%	70.7%	GO:0006955	864	immune response
12	70	748	281	4.96E-07	1.48E-05	23.2%	24.2%	GO:0044260	3935	cellular macromolecule metabolic process
13	36	172	79	6.39E-07	4.48E-08	12.6%	11.4%	GO:0015031	745	protein transport
14	8	259	128	1.45E-06	4.25E-12	14.1%	21.9%	GO:0006996	1213	organelle organization and biogenesis
**15**	**131**	**115**	**44**	**1.46E-06**	**4.67E-04**	**7.3%**	**4.5%**	**GO:0006512**	**466**	**ubiquitin cycle**
16	312	95	29	1.75E-06	3.67E-02	18.1%	13.8%	GO:0044265	370	cellular macromolecule catabolic process
17	44	177	80	2.03E-06	1.92E-07	14.8%	12.5%	GO:0046907	784	intracellular transport
18	39	210	94	2.06E-06	1.08E-07	13.8%	13.8%	GO:0051641	957	cellular localization
19	37	206	93	2.08E-06	7.37E-08	13.9%	12.9%	GO:0051649	936	establishment of cellular localization
20	232	1429	497	4.02E-06	1.05E-02	21.6%	19.1%	GO:0043170	8027	macromolecule metabolic process
21	6	225	143	5.41E-06	2.85E-23	24.3%	65.7%	GO:0002376	1050	immune system process
22	88	74	33	1.74E-05	5.17E-05	22.6%	27.3%	GO:0006457	287	protein folding
23	59	25	15	1.74E-05	1.36E-06	18.8%	40.0%	GO:0009142	69	nucleoside triphosphate biosynthetic process
24	354	55	16	1.81E-05	6.44E-02	19.2%	6.3%	GO:0051603	198	proteolysis involved in cellular protein catabolic process
25	27	30	21	1.81E-05	4.15E-09	28.1%	57.1%	GO:0006752	89	group transfer coenzyme metabolic process
26	275	109	36	1.86E-05	2.48E-02	15.9%	13.9%	GO:0009057	460	macromolecule catabolic process
27	359	55	16	2.12E-05	6.69E-02	19.1%	6.3%	GO:0044257	199	cellular protein catabolic process
28.5	62.5	24	14	2.27E-05	3.79E-06	19.7%	42.9%	GO:0009145	66	purine nucleoside triphosphate biosynthetic process
28.5	62.5	24	14	2.27E-05	3.79E-06	19.7%	42.9%	GO:0009206	66	purine ribonucleoside triphosphate biosynthetic process
30	51	28	17	2.41E-05	7.19E-07	19.5%	35.3%	GO:0009141	82	nucleoside triphosphate metabolic process
31.5	401	54	15	2.72E-05	1.00E-01	19.4%	6.7%	GO:0019941	196	modification-dependent protein catabolic process
31.5	401	54	15	2.72E-05	1.00E-01	19.4%	6.7%	GO:0043632	196	modification-dependent macromolecule catabolic process
33	64	24	14	3.06E-05	4.62E-06	19.4%	42.9%	GO:0009201	67	ribonucleoside triphosphate biosynthetic process
34	55.5	26	16	3.16E-05	9.02E-07	20.0%	37.5%	GO:0009144	75	purine nucleoside triphosphate metabolic process
35	230	412	145	3.41E-05	1.00E-02	21.4%	22.1%	GO:0043412	2115	biopolymer modification
36	29	31	21	3.66E-05	1.87E-08	24.0%	28.6%	GO:0009150	96	purine ribonucleotide metabolic process
37	392	53	15	4.76E-05	9.68E-02	19.5%	6.7%	GO:0006511	195	ubiquitin-dependent protein catabolic process
38	40	29	19	5.00E-05	1.16E-07	23.6%	31.6%	GO:0009152	89	purine ribonucleotide biosynthetic process
**39**	**10**	**54**	**37**	**5.02E-05**	**5.77E-11**	**7.5%**	**5.4%**	**GO:0022613**	**200**	**ribonucleoprotein complex biogenesis and assembly**
40	53	25	16	7.07E-05	7.36E-07	20.3%	37.5%	GO:0009205	74	purine ribonucleoside triphosphate metabolic process
41.5	65.5	8	6	7.81E-05	4.89E-06	28.6%	50.0%	GO:0006098	14	pentose-phosphate shunt
41.5	65.5	8	6	7.81E-05	4.89E-06	28.6%	50.0%	GO:0006740	14	NADPH regeneration
43	28	32	22	8.44E-05	1.83E-08	22.1%	27.3%	GO:0006163	104	purine nucleotide metabolic process
44	50	104	52	8.94E-05	5.93E-07	15.1%	11.5%	GO:0006886	451	intracellular protein transport
45	55.5	25	16	9.15E-05	9.02E-07	20.0%	37.5%	GO:0009199	75	ribonucleoside triphosphate metabolic process
46	38	30	20	9.49E-05	9.26E-08	21.9%	30.0%	GO:0006164	96	purine nucleotide biosynthetic process
47	358	65	20	9.53E-05	6.67E-02	15.6%	10.0%	GO:0030163	257	protein catabolic process
48	211	394	142	1.14E-04	6.56E-03	21.5%	22.5%	GO:0006464	2041	protein modification process
49	9	12	13	1.26E-04	3.94E-11	63.0%	76.9%	GO:0009396	27	folic acid and derivative biosynthetic process
50	21	56	37	1.45E-04	6.80E-10	25.3%	56.8%	GO:0006732	217	coenzyme metabolic process
51	311	1058	367	1.58E-04	3.53E-02	20.3%	17.7%	GO:0043283	5927	biopolymer metabolic process
52	16.5	10	11	1.62E-04	2.23E-10	42.9%	63.6%	GO:0007019	21	microtubule depolymerization
53	84	9	6	1.72E-04	3.70E-05	22.2%	50.0%	GO:0006739	18	NADP metabolic process
54	52	30	19	1.79E-04	7.28E-07	23.2%	31.6%	GO:0009260	99	ribonucleotide biosynthetic process
55	283	334	116	1.82E-04	2.69E-02	21.6%	21.6%	GO:0043687	1713	post-translational protein modification
56	286	82	28	1.86E-04	2.81E-02	13.8%	7.1%	GO:0043285	347	biopolymer catabolic process
57	43	32	21	1.89E-04	1.76E-07	23.1%	28.6%	GO:0009259	108	ribonucleotide metabolic process
58	18	39	29	2.12E-04	2.92E-10	30.0%	62.1%	GO:0009108	140	coenzyme biosynthetic process

Rank		Hits		Signifincance		Err%		Gene set info		
eGSA	IGA	eGSA	IGA	eGSA	IGA	Total	Hits	GO id	Gene #	Title
59	281	1608	562	2.16E-04	2.67E-02	21.9%	20.5%	GO:0044238	9257	primary metabolic process
60	210	1600	568	2.32E-04	6.46E-03	22.0%	21.8%	GO:0044237	9211	cellular metabolic process
61	49	50	29	2.36E-04	5.57E-07	6.3%	10.3%	GO:0006605	192	protein targeting
62	25	63	40	2.42E-04	2.07E-09	25.1%	52.5%	GO:0051186	255	cofactor metabolic process
63	100	10	6	2.77E-04	1.62E-04	36.4%	66.7%	GO:0009147	22	pyrimidine nucleoside triphosphate metabolic process
64	68	127	60	3.38E-04	5.35E-06	17.2%	21.7%	GO:0006412	587	translation
65.5	143	16	8	3.44E-04	7.59E-04	13.6%	25.0%	GO:0015985	44	energy coupled proton transport, down electrochemical gradient
65.5	143	16	8	3.44E-04	7.59E-04	13.6%	25.0%	GO:0015986	44	ATP synthesis coupled proton transport
67.5	168	17	8	3.58E-04	1.46E-03	12.5%	25.0%	GO:0006753	48	nucleoside phosphate metabolic process
67.5	168	17	8	3.58E-04	1.46E-03	12.5%	25.0%	GO:0006754	48	ATP biosynthetic process
69	23	13	13	3.67E-04	1.16E-09	54.5%	76.9%	GO:0006760	33	folic acid and derivative metabolic process
70	20	231	117	4.30E-04	5.04E-10	18.0%	34.2%	GO:0044249	1156	cellular biosynthetic process
71.5	11.5	9	11	5.28E-04	1.01E-10	45.0%	63.6%	GO:0007026	20	negative regulation of microtubule depolymerization
71.5	11.5	9	11	5.28E-04	1.01E-10	45.0%	63.6%	GO:0031114	20	regulation of microtubule depolymerization
73	24	42	30	5.36E-04	2.05E-09	29.4%	60.0%	GO:0051188	160	cofactor biosynthetic process
74	67	59	32	8.11E-04	5.05E-06	24.0%	34.4%	GO:0007017	246	microtubule-based process
75	16.5	9	11	8.60E-04	2.23E-10	42.9%	63.6%	GO:0031111	21	negative regulation of microtubule polymerization or depolymerization
**76**	**35**	**26**	**20**	**9.52E-04**	**2.80E-08**	**6.7%**	**0.0%**	**GO:0042254**	**90**	**ribosome biogenesis and assembly**
77	185	105	42	9.67E-04	3.23E-03	10.1%	0.0%	GO:0006396	485	RNA processing
78	121	18	10	1.05E-03	2.95E-04	14.3%	20.0%	GO:0046034	56	ATP metabolic process
**79.5**	**175**	**10**	**5**	**1.08E-03**	**2.45E-03**	**0.0%**	**0.0%**	**GO:0007093**	**25**	**mitotic cell cycle checkpoint**
79.5	26	10	11	1.08E-03	3.18E-09	36.0%	63.6%	GO:0031109	25	microtubule polymerization or depolymerization
81	202	11	5	1.25E-03	5.41E-03	13.8%	20.0%	GO:0006612	29	protein targeting to membrane
82	90	180	79	1.32E-03	5.58E-05	8.5%	12.7%	GO:0007049	896	cell cycle
83	19	9	11	1.35E-03	4.65E-10	40.9%	63.6%	GO:0031110	22	regulation of microtubule polymerization or depolymerization
84	42	59	36	1.51E-03	1.42E-07	19.4%	27.8%	GO:0009117	252	nucleotide metabolic process
85	82	178	80	1.81E-03	2.68E-05	16.8%	25.0%	GO:0009059	891	macromolecule biosynthetic process
86.5	102	7	5	1.94E-03	1.73E-04	43.8%	80.0%	GO:0006228	16	UTP biosynthetic process
86.5	102	7	5	1.94E-03	1.73E-04	43.8%	80.0%	GO:0046051	16	UTP metabolic process
88	138	154	65	2.14E-03	5.68E-04	7.7%	10.8%	GO:0022402	762	cell cycle process
**89**	**247**	**5**	**2**	**2.25E-03**	**1.68E-02**	**10.0%**	**0.0%**	**GO:0006213**	**10**	**pyrimidine nucleoside metabolic process**
90	69	10	8	2.30E-03	1.21E-05	25.9%	50.0%	GO:0006769	27	nicotinamide metabolic process
91	31	39	28	2.43E-03	2.08E-08	24.2%	32.1%	GO:0006399	157	tRNA metabolic process
92	120	13	8	2.56E-03	2.94E-04	25.6%	50.0%	GO:0006220	39	pyrimidine nucleotide metabolic process
**93**	**217**	**28**	**12**	**2.59E-03**	**7.16E-03**	**3.8%**	**0.0%**	**GO:0043122**	**105**	**regulation of I-kappaB kinase/NF-kappaB cascade**
**94**	**216**	**18**	**8**	**2.61E-03**	**6.97E-03**	**3.3%**	**0.0%**	**GO:0007088**	**60**	**regulation of mitosis**
95	105	115	52	2.96E-03	2.01E-04	7.7%	11.5%	GO:0000074	555	regulation of progression through cell cycle
**96**	**95**	**32**	**18**	**3.05E-03**	**9.40E-05**	**8.8%**	**5.6%**	**GO:0048193**	**125**	**Golgi vesicle transport**
98	14	23	22	3.05E-03	1.56E-10	28.9%	40.9%	GO:0006418	83	tRNA aminoacylation for protein translation
98	14	23	22	3.05E-03	1.56E-10	28.9%	40.9%	GO:0043038	83	amino acid activation
98	14	23	22	3.05E-03	1.56E-10	28.9%	40.9%	GO:0043039	83	tRNA aminoacylation
100	115	116	52	3.07E-03	2.62E-04	8.0%	11.5%	GO:0051726	561	regulation of cell cycle

B

Rank		Hits		Signifincance		Err%		Gene set info		
eGSA	IGA	eGSA	IGA	eGSA	IGA	Total	Hits	GO id	Gene #	Title
1	1	2715	994	0	0	25.5%	23.9%	GO:0008150	18033	biological_process
2	12	166	73	5.61E-12	7.10E-09	22.9%	21.9%	GO:0006091	668	generation of precursor metabolites and energy
3	10	133	60	5.99E-12	5.16E-09	27.7%	26.7%	GO:0006118	502	electron transport
4	7	152	74	3.45E-11	4.82E-11	17.7%	16.2%	GO:0006082	610	organic acid metabolic process
5	6	151	74	5.19E-11	4.12E-11	17.8%	16.2%	GO:0019752	608	carboxylic acid metabolic process
6	25	35	16	3.62E-10	1.90E-06	15.9%	25.0%	GO:0044270	82	nitrogen compound catabolic process
7	22	34	16	7.11E-10	1.32E-06	16.3%	25.0%	GO:0009310	80	amine catabolic process
8	26	31	15	5.38E-09	2.11E-06	17.6%	26.7%	GO:0009063	74	amino acid catabolic process
9	15	122	56	4.39E-08	3.37E-07	17.2%	21.4%	GO:0006807	512	nitrogen compound metabolic process
10	14	114	55	1.77E-07	1.04E-07	17.6%	21.8%	GO:0009308	482	amine metabolic process
11	20	97	46	3.05E-07	6.93E-07	19.1%	21.7%	GO:0006519	398	amino acid and derivative metabolic process
12	27	82	39	9.65E-07	2.43E-06	20.0%	23.1%	GO:0006520	330	amino acid metabolic process
13	2	140	83	1.03E-06	7.90E-14	13.3%	16.9%	GO:0044255	639	cellular lipid metabolic process
14	3	168	96	1.63E-06	1.34E-13	14.6%	17.7%	GO:0006629	800	lipid metabolic process
15	30	64	31	3.24E-06	6.67E-06	13.3%	6.5%	GO:0032787	248	monocarboxylic acid metabolic process
16	8	16	13	4.84E-06	2.65E-09	25.0%	23.1%	GO:0009069	36	serine family amino acid metabolic process
17.5	23.5	17	11	1.38E-05	1.75E-06	14.3%	9.1%	GO:0002541	42	activation of plasma proteins during acute inflammatory response
17.5	23.5	17	11	1.38E-05	1.75E-06	14.3%	9.1%	GO:0006956	42	complement activation
**19**	**38**	**26**	**13**	**1.42E-05**	**8.19E-05**	**9.0%**	**7.7%**	**GO:0002526**	**78**	**acute inflammatory response**
20	13	8	7	1.57E-05	8.33E-08	38.5%	42.9%	GO:0006546	13	glycine catabolic process
21	16	12	9	1.73E-05	3.79E-07	36.0%	33.3%	GO:0006544	25	glycine metabolic process
**22**	**19**	**45**	**26**	**2.60E-05**	**5.10E-07**	**6.5%**	**7.7%**	**GO:0006631**	**168**	**fatty acid metabolic process**
23	11	8	8	3.82E-05	7.06E-09	35.7%	37.5%	GO:0009071	14	serine family amino acid catabolic process
**24**	**17**	**117**	**60**	**1.07E-04**	**4.03E-07**	**3.4%**	**3.3%**	**GO:0009605**	**566**	**response to external stimulus**
25	98.5	9	4	1.47E-04	3.06E-03	15.8%	50.0%	GO:0000051	19	urea cycle intermediate metabolic process
26	9	78	48	1.93E-04	3.05E-09	17.4%	14.6%	GO:0006066	356	alcohol metabolic process
**27**	**21**	**85**	**46**	**2.89E-04**	**7.45E-07**	**4.3%**	**4.3%**	**GO:0009611**	**399**	**response to wounding**
**28**	**270**	**7**	**2**	**3.34E-04**	**3.85E-02**	**7.1%**	**0.0%**	**GO:0009081**	**14**	**branched chain family amino acid metabolic process**
**29**	**18**	**22**	**16**	**3.75E-04**	**4.08E-07**	**1.4%**	**0.0%**	**GO:0008203**	**74**	**cholesterol metabolic process**
**30**	**41.5**	**11**	**7**	**3.80E-04**	**9.57E-05**	**3.6%**	**0.0%**	**GO:0006958**	**28**	**complement activation, classical pathway**
31	107	18	8	4.46E-04	3.74E-03	22.8%	37.5%	GO:0009064	57	glutamine family amino acid metabolic process
32	4	47	40	4.93E-04	1.61E-13	16.6%	25.0%	GO:0008202	199	steroid metabolic process
33	91	8	4	5.22E-04	2.36E-03	16.7%	50.0%	GO:0006525	18	arginine metabolic process
**34.5**	**103**	**6**	**3**	**6.84E-04**	**3.18E-03**	**0.0%**	**0.0%**	**GO:0030193**	**12**	**regulation of blood coagulation**
**34.5**	**103**	**6**	**3**	**6.84E-04**	**3.18E-03**	**0.0%**	**0.0%**	**GO:0030195**	**12**	**negative regulation of blood coagulation**
**36**	**47**	**11**	**7**	**8.06E-04**	**1.63E-04**	**3.3%**	**0.0%**	**GO:0002455**	**30**	**humoral immune response mediated by circulating immunoglobulin**
38	59	8	5	8.60E-04	4.03E-04	21.1%	40.0%	GO:0006081	19	aldehyde metabolic process
38	98.5	8	4	8.60E-04	3.06E-03	15.8%	0.0%	GO:0019439	19	aromatic compound catabolic process
38	28	8	7	8.60E-04	3.63E-06	5.3%	14.3%	GO:0006635	19	fatty acid beta-oxidation
**40**	**37**	**36**	**20**	**9.01E-04**	**5.66E-05**	**2.7%**	**5.0%**	**GO:0008015**	**147**	**circulation**
41	5	24	24	9.51E-04	5.45E-12	8.0%	25.0%	GO:0016125	88	sterol metabolic process
42	109	15	7	9.52E-04	3.83E-03	4.3%	14.3%	GO:0008217	47	blood pressure regulation
43	33	22	14	1.04E-03	2.31E-05	8.9%	14.3%	GO:0006959	79	humoral immune response
**44**	**69**	**26**	**13**	**1.20E-03**	**1.04E-03**	**9.1%**	**7.7%**	**GO:0007596**	**99**	**blood coagulation**
**45**	**62**	**31**	**16**	**1.24E-03**	**4.58E-04**	**8.1%**	**6.3%**	**GO:0050878**	**124**	**regulation of body fluids**
**46**	**259**	**6**	**2**	**1.29E-03**	**3.15E-02**	**7.7%**	**0.0%**	**GO:0042219**	**13**	**amino acid derivative catabolic process**
48.5	82.5	5	3	1.41E-03	1.47E-03	30.0%	66.7%	GO:0006526	10	arginine biosynthetic process
**48.5**	**44**	**5**	**4**	**1.41E-03**	**1.01E-04**	**0.0%**	**0.0%**	**GO:0016054**	**10**	**organic acid catabolic process**
**48.5**	**44**	**5**	**4**	**1.41E-03**	**1.01E-04**	**0.0%**	**0.0%**	**GO:0046395**	**10**	**carboxylic acid catabolic process**
**48.5**	**422**	**5**	**1**	**1.41E-03**	**1.02E-01**	**10.0%**	**0.0%**	**GO:0042402**	**10**	**biogenic amine catabolic process**
**51**	**58**	**30**	**16**	**1.44E-03**	**3.10E-04**	**7.5%**	**6.3%**	**GO:0042060**	**120**	**wound healing**
52	41.5	10	7	1.58E-03	9.57E-05	3.6%	14.3%	GO:0019395	28	fatty acid oxidation
53	155	17	7	1.59E-03	1.37E-02	25.9%	28.6%	GO:0008652	58	amino acid biosynthetic process
**54**	**76**	**26**	**13**	**1.66E-03**	**1.27E-03**	**8.9%**	**7.7%**	**GO:0050817**	**101**	**coagulation**
55	34	20	13	1.98E-03	3.82E-05	13.7%	7.7%	GO:0002253	73	activation of immune response
56	63	26	14	2.26E-03	5.09E-04	10.7%	7.1%	GO:0051240	103	positive regulation of multicellular organismal process
57	88	38	18	2.34E-03	1.89E-03	31.5%	22.2%	GO:0006725	165	aromatic compound metabolic process
58	581	189	64	2.40E-03	1.65E-01	29.4%	23.4%	GO:0006811	1044	ion transport
**59**	**86**	**26**	**13**	**2.62E-03**	**1.69E-03**	**8.7%**	**7.7%**	**GO:0007599**	**104**	**hemostasis**
60	1023	5	0	2.70E-03	4.64E-01	45.5%	0.0%	GO:0015780	11	nucleotide-sugar transport
61.5	56.5	22	13	2.99E-03	2.12E-04	11.8%	7.7%	GO:0002684	85	positive regulation of immune system process
61.5	56.5	22	13	2.99E-03	2.12E-04	11.8%	7.7%	GO:0050778	85	positive regulation of immune response
63	376	10	3	3.01E-03	8.06E-02	40.0%	66.7%	GO:0009084	30	glutamine family amino acid biosynthetic process
**64**	**132**	**22**	**10**	**3.51E-03**	**7.49E-03**	**2.3%**	**0.0%**	**GO:0045087**	**86**	**innate immune response**
**65.5**	**134**	**6**	**3**	**3.67E-03**	**7.68E-03**	**0.0%**	**0.0%**	**GO:0050818**	**15**	**regulation of coagulation**
**65.5**	**134**	**6**	**3**	**3.67E-03**	**7.68E-03**	**0.0%**	**0.0%**	**GO:0050819**	**15**	**negative regulation of coagulation**
**67**	**620**	**9**	**2**	**4.24E-03**	**1.84E-01**	**3.7%**	**0.0%**	**GO:0042133**	**27**	**neurotransmitter metabolic process**
68	73	59	28	4.53E-03	1.23E-03	10.1%	10.7%	GO:0008610	287	lipid biosynthetic process
70	233	5	2	4.72E-03	2.53E-02	16.7%	0.0%	GO:0008209	12	androgen metabolic process
**70**	**103**	**5**	**3**	**4.72E-03**	**3.18E-03**	**0.0%**	**0.0%**	**GO:0006957**	**12**	**complement activation, alternative pathway**
70	31.5	5	5	4.72E-03	1.92E-05	25.0%	0.0%	GO:0006563	12	L-serine metabolic process

Rank		Hits		Signifincance		Err%		Gene set info		
eGSA	IGA	eGSA	IGA	eGSA	IGA	Total	Hits	GO id	Gene #	Title
**72**	**139**	**15**	**7**	**4.74E-03**	**9.03E-03**	**1.9%**	**0.0%**	**GO:0007588**	**54**	**excretion**
73.5	69	24	13	5.44E-03	1.04E-03	10.1%	7.7%	GO:0002682	99	regulation of immune system process
73.5	69	24	13	5.44E-03	1.04E-03	10.1%	7.7%	GO:0050776	99	regulation of immune response
75	119	25	12	6.13E-03	5.07E-03	19.0%	16.7%	GO:0044271	105	nitrogen compound biosynthetic process
**76**	**93.5**	**19**	**10**	**6.44E-03**	**2.59E-03**	**6.7%**	**0.0%**	**GO:0042445**	**75**	**hormone metabolic process**
**77**	**60**	**20**	**12**	**6.47E-03**	**4.05E-04**	**3.8%**	**0.0%**	**GO:0006694**	**80**	**steroid biosynthetic process**
79	259	5	2	7.65E-03	3.15E-02	15.4%	0.0%	GO:0006536	13	glutamate metabolic process
79	259	5	2	7.65E-03	3.15E-02	23.1%	50.0%	GO:0006547	13	histidine metabolic process
79	259	5	2	7.65E-03	3.15E-02	23.1%	50.0%	GO:0009075	13	histidine family amino acid metabolic process
**81**	**40**	**57**	**31**	**7.96E-03**	**9.37E-05**	**2.8%**	**3.2%**	**GO:0006954**	**283**	**inflammatory response**
**82**	**90**	**8**	**5**	**8.13E-03**	**1.98E-03**	**0.0%**	**0.0%**	**GO:0006695**	**25**	**cholesterol biosynthetic process**
83	95	21	11	8.61E-03	2.86E-03	23.0%	18.2%	GO:0009309	87	amine biosynthetic process
**84**	**84.5**	**24**	**13**	**9.24E-03**	**1.54E-03**	**7.8%**	**7.7%**	**GO:0002252**	**103**	**immune effector process**
85.5	215	9	4	9.85E-03	2.28E-02	20.0%	0.0%	GO:0019751	30	polyol metabolic process
**85.5**	**696**	**9**	**2**	**9.85E-03**	**2.28E-01**	**3.3%**	**0.0%**	**GO:0006953**	**30**	**acute-phase response**
**88.5**	**82.5**	**4**	**3**	**1.00E-02**	**1.47E-03**	**10.0%**	**0.0%**	**GO:0006206**	**10**	**pyrimidine base metabolic process**
**88.5**	**165**	**4**	**2**	**1.00E-02**	**1.50E-02**	**10.0%**	**0.0%**	**GO:0006558**	**10**	**L-phenylalanine metabolic process**
**88.5**	**165**	**4**	**2**	**1.00E-02**	**1.50E-02**	**10.0%**	**0.0%**	**GO:0006559**	**10**	**L-phenylalanine catabolic process**
88.5	44	4	4	1.00E-02	1.01E-04	40.0%	50.0%	GO:0030212	10	hyaluronan metabolic process
**91**	**89**	**56**	**27**	**1.12E-02**	**1.92E-03**	**5.7%**	**3.7%**	**GO:0051239**	**282**	**regulation of multicellular organismal process**
92	468	7	2	1.16E-02	1.18E-01	45.5%	50.0%	GO:0015671	22	oxygen transport
**94**	**126**	**5**	**3**	**1.17E-02**	**5.89E-03**	**0.0%**	**0.0%**	**GO:0009595**	**14**	**detection of biotic stimulus**
**94**	**605**	**5**	**1**	**1.17E-02**	**1.79E-01**	**0.0%**	**0.0%**	**GO:0017157**	**14**	**regulation of exocytosis**
**94**	**270**	**5**	**2**	**1.17E-02**	**3.85E-02**	**7.1%**	**0.0%**	**GO:0042311**	**14**	**vasodilation**
96	751	6	1	1.20E-02	2.61E-01	11.1%	0.0%	GO:0009065	18	glutamine family amino acid catabolic process
**97**	**247**	**9**	**4**	**1.26E-02**	**2.59E-02**	**9.7%**	**0.0%**	**GO:0050880**	**31**	**regulation of blood vessel size**
98	184	111	45	1.33E-02	1.94E-02	16.6%	15.6%	GO:0044248	610	cellular catabolic process
**99**	**39**	**524**	**222**	**1.50E-02**	**8.84E-05**	**5.1%**	**5.4%**	**GO:0032501**	**3217**	**multicellular organismal process**
100	154	19	9	1.54E-02	1.33E-02	14.8%	11.1%	GO:0006575	81	amino acid derivative metabolic process
