# Supplementary material for: A novel regulatory event-based gene set analysis method for exploring global functional changes in heterogeneous genomic data sets
Source: BMC Genomics. 2009 Jan 16;10:26. doi: 10.1186/1471-2164-10-26 (PMC2637897; doi:10.1186/1471-2164-10-26)
Supplement: Additional file 3 — Cross-analysis of high regulatory event frequency genes between gene sets and KEGG pathway. The overlapped gene numbers are shown in the "Hits" column and the pathways they are involved in are listed. [file 1471-2164-10-26-S3.pdf]

inflammatory response(GO:0006954:283 genes)

Hits	Pathway
68	Cytokinecytokine receptor interaction(hsa04060)
35	Complement and coagulation cascades(hsa04610)
30	Toll-like receptor signaling pathway(hsa04620)
15	Neuroactive ligand-receptor interaction(hsa04080)
13	Jak-STAT signaling pathway(hsa04630)
12	MAPK signaling pathway(hsa04010)
10	Apoptosis(hsa04210)
10	Regulation of actin cytoskeleton(hsa04810)
10	Epithelial cell signaling in Helicobacter pylori infection(hsa05120)
9	Hematopoietic cell lineage(hsa04640)
8	Cell adhesion molecules (CAMs)(hsa04514)
7	Arachidonic acid metabolism(hsa00590)
7	T cell receptor signaling pathway(hsa04660)
7	B cell receptor signaling pathway(hsa04662)
7	Leukocyte transendothelial migration(hsa04670)
6	Calcium signaling pathway(hsa04020)
6	TGF - beta signaling pathway(hsa04350)
6	VEGF signaling pathway(hsa04370)
6	Natural killer cell mediated cytotoxicity(hsa04650)
6	Fc epsilon RI signaling pathway(hsa04664)
6	Pancreatic cancer(hsa05212)
6	Chronic myeloid leukemia(hsa05220)

immune effector process(GO:0002252:103 genes)

Hits	Pathway
26	Complement and coagulation cascades(hsa04610)
10	T cell receptor signaling pathway(hsa04660)
9	Cytokinecytokine receptor interaction(hsa04060)
6	Natural killer cell mediated cytotoxicity(hsa04650)

innate immune response(GO:0045087:86 genes)

Hits	Pathway
26	Complement and coagulation cascades(hsa04610)
10	Toll-like receptor signaling pathway(hsa04620)
