# Supplementary material for: A novel regulatory event-based gene set analysis method for exploring global functional changes in heterogeneous genomic data sets
Source: BMC Genomics. 2009 Jan 16;10:26. doi: 10.1186/1471-2164-10-26 (PMC2637897; doi:10.1186/1471-2164-10-26)
Supplement: Additional file 5 — Data set information. * obtained from NCBI GEO [32]. # obtained from ArrayExpress [33]. Data set reference: HCC1 [15], HCC2 [17], Tumor1[35], Tumor3 [36] [file 1471-2164-10-26-S5.pdf]

Dataset (id)	Contributors	Type	Sample id
Normal (GSE3526*)	Roth, R.B.	Liver	GSM80728, GSM80729, GSM80730, GSM80739
		Spleen	GSM80807, GSM80808, GSM80825, GSM80826
HCC1 (GSE6764*)	Wurmbach, E. *et al.*	normal	GSM155919, GSM155926, GSM155927, GSM155928, GSM155947, GSM155948, GSM155961, GSM155964, GSM155988, GSM155989
		cirrhosis	GSM155920, GSM155921, GSM155922, GSM155923, GSM155931, GSM155951, GSM155952, GSM155965, GSM155966, GSM155967, GSM155968, GSM155969, GSM155984
		low-grade dysplasia	GSM155924, GSM155925, GSM155971, GSM155972, GSM155973, GSM155974, GSM155976, GSM155978, GSM155979, GSM155980
		high-grade dysplasia	GSM155929, GSM155930, GSM155970, GSM155975, GSM155977, GSM155981, GSM155982
		very early HCC	GSM155932, GSM155933, GSM155934, GSM155935, GSM155987, GSM155991, GSM155993
		early HCC	GSM155937, GSM155939, GSM155945, GSM155946, GSM155955, GSM155958, GSM155959, GSM155960, GSM155992
		advanced HCC	GSM155938, GSM155940, GSM155941, GSM155942, GSM155963, GSM155985, GSM155990
		very advanced HCC	GSM155936, GSM155943, GSM155944, GSM155949, GSM155950, GSM155953, GSM155954, GSM155956, GSM155957, GSM155962
HCC2 (E-TABM-36#)	Boyault, S *et al.*	Liver	HCVCir, AlcCir, AlcnCir, HBCCir, HBCnCir
		HCC subtype 1	HCC010, HCC018, HCC060, HCC170, HCC237, HCC252
		HCC subtype 2	HCC016, HCC189, HCC245, HCC309, HCC402, HCC438
		HCC subtype 3	HCC205, HCC208, HCC226, HCC229, HCC231, HCC301, HCC304, HCC441
		HCC subtype 4	HCC081, HCC107, HCC154, HCC155, HCC191, HCC195, HCC198, HCC204, HCC206, HCC210, HCC218, HCC253, HCC313, HCC314, HCC322, HCC326, HCC340, HCC358, HCC373, HCC405, HCCZR5
		HCC subtype 5	HCC013, HCC037, HCC121, HCC137, HCC164, HCC230, HCC333, HCC429, HCC433, HCC437
		HCC subtype 6	HCC99, HCC168, HCC197, HCC242, HCC305, HCC320, HCC335, HCC399, HCC432
Tumor1 (GSE4107*)	Hong, Y	Colon	GSM93938, GSM93939, GSM93941, GSM93943, GSM93944, GSM93946, GSM93948, GSM93950, GSM93952, GSM93954
		coloerectal cnacer	GSM93789, GSM93920, GSM93921, GSM93922, GSM93923, GSM93924, GSM93925, GSM93926, GSM93927, GSM93928, GSM93929, GSM93932
Tumor2 (GSE3678*)	Ismael, R	Thyroid	GSM85215, GSM85216, GSM85217, GSM85218, GSM85219, GSM85220, GSM85221
		Thyroid tumors	GSM85222, GSM85223, GSM85224, GSM85225, GSM85226, GSM85227, GSM85228
Tumor3 (GSE7670*)	Su, L *et. al.*	Lung	GSM185811, GSM185813, GSM185815, GSM185817, GSM185819, GSM185821, GSM185823, GSM185825, GSM185827, GSM185829, GSM185831, GSM185833, GSM185835, GSM185837, GSM185839, GSM185841, GSM185843, GSM185845, GSM185847, GSM185849, GSM185851, GSM185853, GSM185855, GSM185857, GSM185859 GSM185861 GSM185863 GSM185865 GSM185867 GSM185868
		Lung cell line	GSM185869, GSM185870, GSM185871, GSM185872, GSM185873, GSM185874, GSM185875, GSM185876
		Lung tumors	GSM185812, GSM185814, GSM185816, GSM185818, GSM185820, GSM185822, GSM185824, GSM185826, GSM185828, GSM185830, GSM185832, GSM185834, GSM185836, GSM185838, GSM185840, GSM185842, GSM185844, GSM185846, GSM185848, GSM185850, GSM185852, GSM185854, GSM185856, GSM185858, GSM185860 GSM185862 GSM185864 GSM185866
